# Modelling and Analysis on Biomechanical Dynamic Characteristics of Knee Flexion Movement under Squatting

**DOI:** 10.1155/2014/321080

**Published:** 2014-06-11

**Authors:** Jianping Wang, Kun Tao, Huanyi Li, Chengtao Wang

**Affiliations:** ^1^School of Mechanical Engineering, Henan Polytechnic University, Jiaozuo 454003, China; ^2^Department of Orthopedics, Shanghai 10th People's Hospital, Tongji University School of Medicine, Shanghai 200072, China; ^3^Department of Orthopedics, Zhenjiang Jinshan Hospital, Zhenjiang 212001, China; ^4^School of Mechanical Engineering, Shanghai Jiao Tong University, Shanghai 200240, China

## Abstract

The model of three-dimensional (3D) geometric knee was built, which included femoral-tibial, patellofemoral articulations and the bone and soft tissues. Dynamic finite element (FE) model of knee was developed to simulate both the kinematics and the internal stresses during knee flexion. The biomechanical experimental system of knee was built to simulate knee squatting using cadaver knees. The flexion motion and dynamic contact characteristics of knee were analyzed, and verified by comparing with the data from in vitro experiment. The results showed that the established dynamic FE models of knee are capable of predicting kinematics and the contact stresses during flexion, and could be an efficient tool for the analysis of total knee replacement (TKR) and knee prosthesis design.

## 1. Introduction

All kinds of movements of the knee joint are harmonious in each joint. Due to the complicated structure and large quantity of motion, its noneffective rate in all the joints is on top. The success rate of artificial joint replacement surgery has reached 90% [1]. Even so, there were functional failure, prosthesis loosening or dislocation and the excessive wear of prosthesis, and so forth, postoperatively [2, 3]. The main factors for operation failure, in addition to the pathological reasons, come from operative and prosthetic aspects. However, the disease prevention of human knee, the design of artificial knee prosthesis, and the improvement of surgical technique depended on the research into the movement, stress, and such biomechanical characteristics about natural and artificial knee joint [4].

The internal joint contact stress and distribution of natural and artificial knee joint are directly related to its functional activities. It becomes more important to analyze the biomechanical characteristics of the motion and stress during high flexion activities [5], especially significant for population groups where lifestyle and work activities or religious activities demand deep flexion such as squatting and kneeling. However, the corresponding measurement and prediction are relatively difficult because of the limitation of ethic and measuring devices. So far, there were few effective methods to directly measure the internal stress and distribution for in vivo knee. Therefore, establishing the knee joint calculation model becomes a widely used method for predicting the internal stress and distribution. Among them, the dynamic finite element analysis has been developed into an effective method to predict the internal stress and distribution under dynamic loading conditions [6–9]. In previous models, relatively small range of flexion was conducted, usually not more than 120 degrees; there were less finite element models which can simultaneously proceed with dynamic synchronous prediction for the patellofemoral joint and femorotibial joint [7], and less have represented the whole joint with physiological soft tissue constraint [10].

In this paper, the anatomical model of three-dimensional geometric natural knee was reconstructed. Dynamic finite element (FE) model of natural knee joint, which includes tibiofemoral, patellofemoral articulations and the surrounding soft tissues, was developed in this research, to simulate both the kinematics and internal stress during knee flexion. The biomechanical experimental system of knee flexion motion was set up to simulate human knee squatting using cadaver knees. The flexion motion and dynamic contact characteristics of knee joint were analyzed and were verified by comparison with the data from cadaver in vitro experiment.

## 2. Materials and Methods

### 2.1. Dynamic FE Knee Model

#### 2.1.1. The Modeling of Geometric Knee

**Figure 1:**
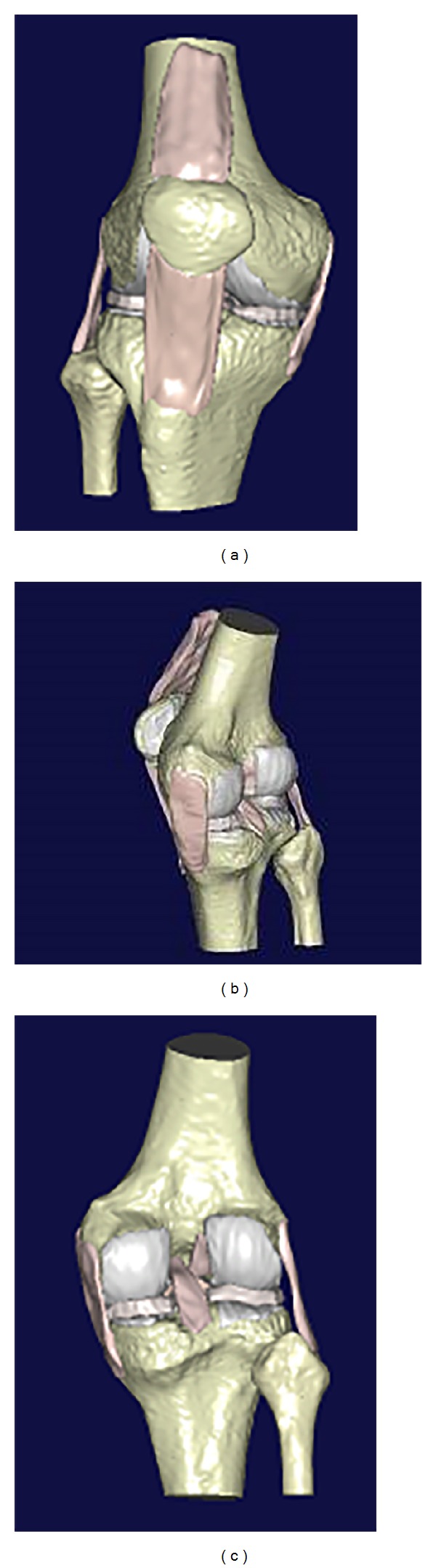
The geometric anatomy model of human knee.

The knee of a healthy volunteer (height 1.73 m, weight 60 kg, male) had been scanned by CT (computed tomography) and MRI (magnetic resonance imaging), respectively. Then the simulation models of the knee joint bone and soft tissues were reconstructed, respectively. Due to the errors of reconstruction leading to the errors of measurement and calculation of motion, the research of precision of cortical bone reconstruction had been carried out [11]. However, the extraction of CT and MRI data was in different coordinates. It could not be directly obtained for geometric anatomic model of knee, which includes both bone and soft tissue. Bone's point cloud contour with clear geometric feature point can be cached through the MRI, so the principle of three-dimensional image registration [12] was adopted to register the point cloud contour obtained by MRI and the bone tissue profile obtained by CT. Then, a complete geometry simulation model of knee joint was built by registration of soft and bone tissues (Figure 1). The methodology and models used in this work were described thoroughly by Wang et al. (2009) [13].

#### 2.1.2. The FE Model of Knee

**Figure 2:**
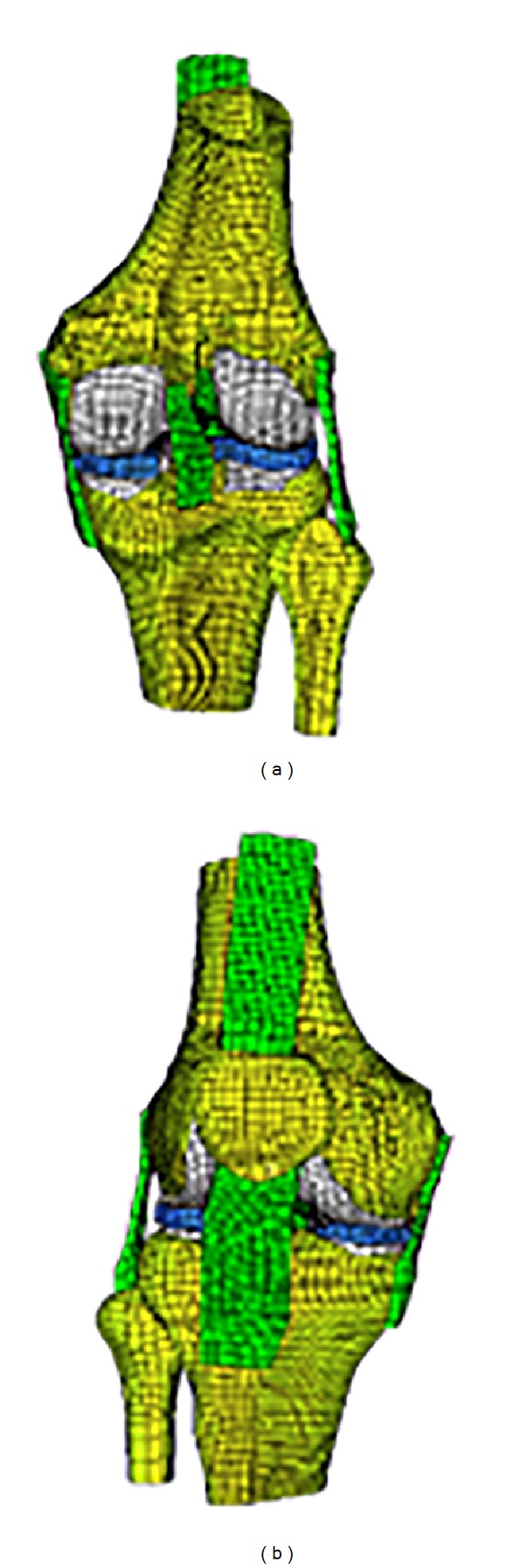
The FE model of knee.

Based on the above anatomical model, the mesh model of natural knee was established, which includes both bone and soft tissues, such as cartilage, meniscus, anterior and posterior cruciate ligament, medial and lateral collateral ligaments, and patellar tendon (Figure 2). The hexahedron units were adopted in all bone and soft tissues of knee FE model to reduce calculation cost. The methodology and models used in this work were described thoroughly by Wang et al. (2009) [14].

There are different features of each tissue's material properties. The material properties of the different tissues derived from literatures facilitate comparison with them. For MCL and LCL, it is an average constant from Gardiner et al. (2001) [15]. For ACL, it is single axial stress strain curve from Butler et al. (1980) [16]. For cruciate ligament and patellar tendon, it was from Suggs et al. (2008) [17]. The meniscus can be regarded as elastic and isotropic in axial, radial, and circumferential direction, respectively [18]. In this paper, the meniscus material parameters were from LeRoux and Setton (2002) [19].

Load and boundary conditions, for the FE model of natural knee, were consistent with the experimental conditions. The value of 400 N was applied to the quadriceps, which was parallel to the femur shaft and directed to the starting point of quadriceps. The value of 300 N force was applied along with the knee joint force line to simulate the weight of body [20]. Under the control of the applied muscles force, the femur move is relative to the tibia with full freedom. The movement of tibia was not active and it was* determined* by the loading conditions distributed in knee model. Nine surface contact pairs were defined for the femur, tibia, patella, and other soft tissue.

### 2.2. Experimental Verification

**Figure 3:**
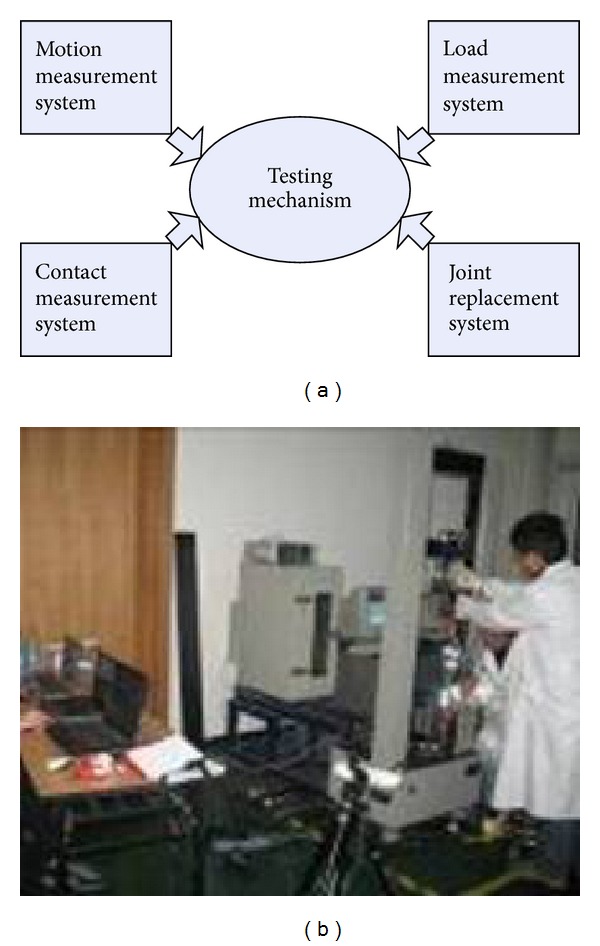
The flowchart and the real of knee experiment system.

Six male volunteers' lower limb specimens were mounted into the specially designed loading and connecting device, which were connected with the standard material testing machine (CSS-44010, Changchun Research Institute for Testing machines Co., Ltd, China). This measurement system was adopted to measure both the dynamic movement and contact stress of both the tibiofemoral and the patellofemoral joints (Figure 3).

#### 2.2.1. Experimental Devices

**Figure 4:**
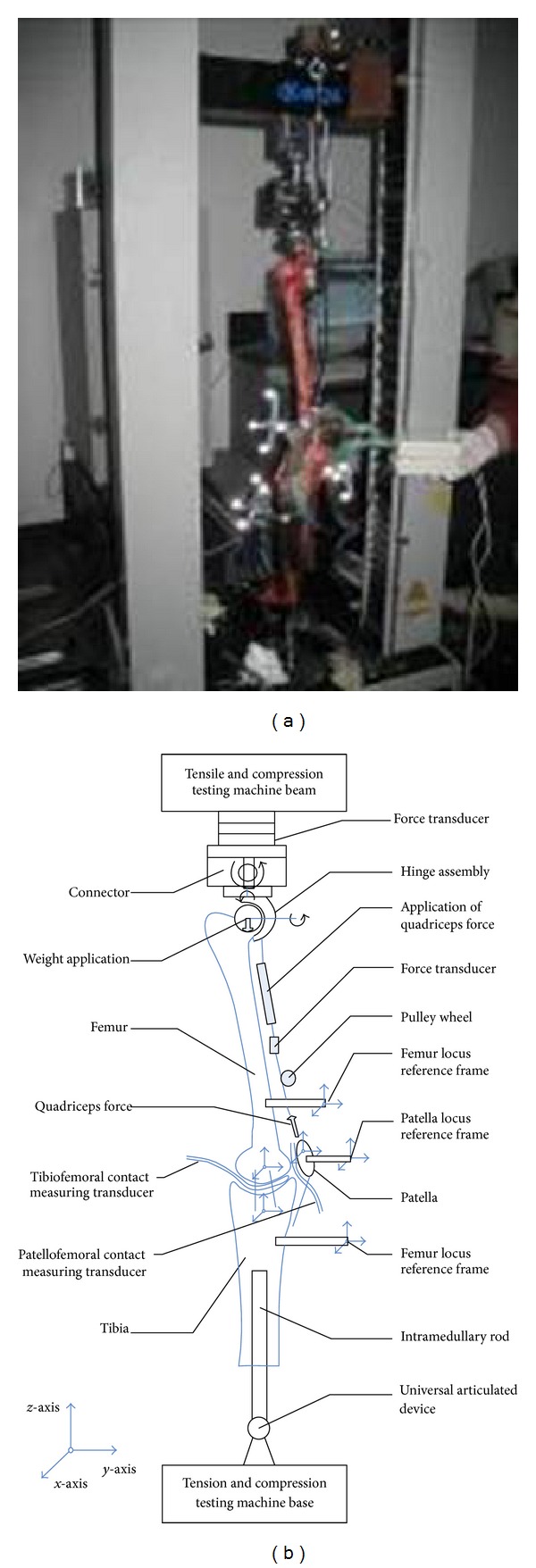
Setup of knee experiment.

To realize simulation of squat movement and force loading, loading and connecting mechanisms were designed connected with the tension/compression testing machine, based on the biomechanical and boundary conditions of FEA. Accompanying squatting, three sets of measuring system were used to measure* synchronously* the relative motion and the stress of tibiofemoral and patellofemoral joint, realizing force control and loading. Experimental devices were composed of devices of loading and connective devices and the tension/compression testing machine. The loading and connective devices were designed to connect the lower limbs of cadavers and tension/compression testing machine, loading the gravity and quadriceps force to realize joint flexion, which were composed of the upper and the lower connection device (Figure 4). The cadaver knee's dynamic translation and rotation (and lock) in coronal, sagittal, and cross sectional planes can be implemented, simulating living knee movement.

**Figure 5:**
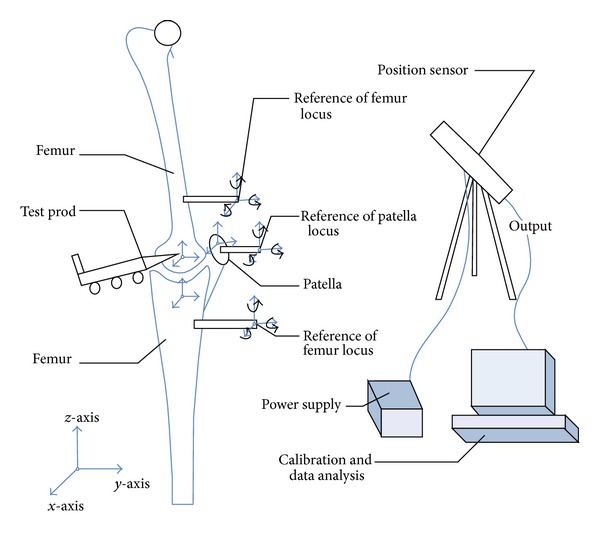
The measurement system of movement.

Optical tracking system (Polaris hybrid optical tracking system, NDI, Calgary, AB, Canada) was used to measure the trajectory of the femur, tibia, and patella under squatting for analysis of knee relative motion by coordinate transformation [21] (Figure 5). Coordinate system was established by measuring knee's bone marker points, and knee joint rotations were defined according to the clinical joint coordinate system (JCS) [22, 23]. The same method was used for FE model.

**Figure 6:**
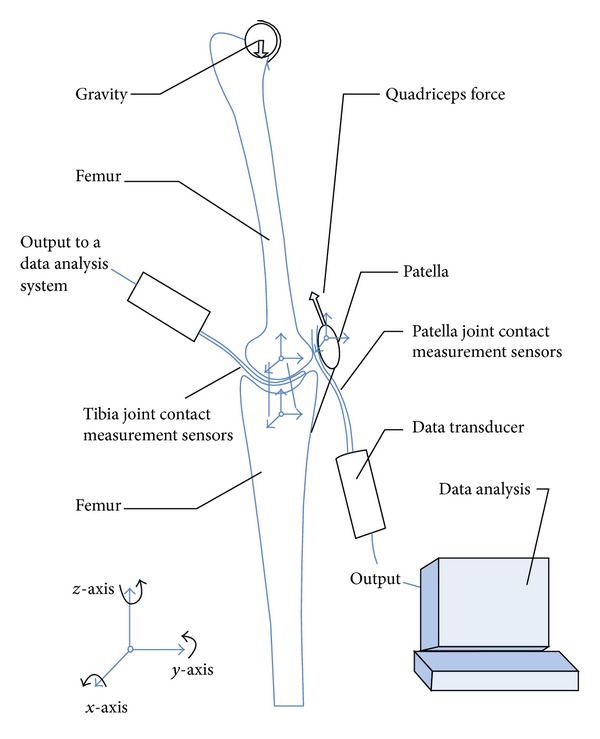
The measurement system of contact.

**Figure 7:**
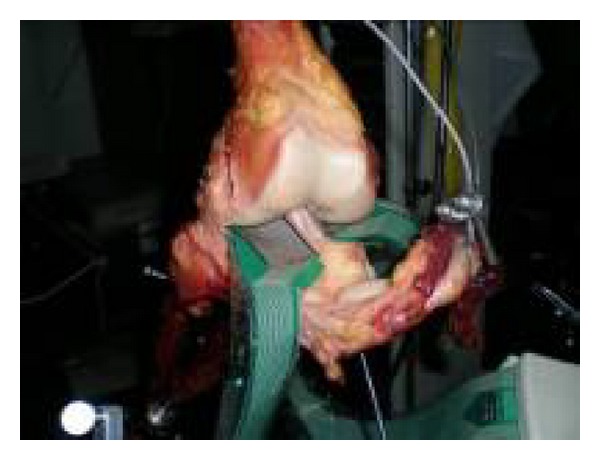
The location of Tecscan sensor.

Tecscan measurement system (Tescan Inc., Boston, MA, USA) was used to implement contact measurement (Figure 6). The measurement system is comprised of I-scan sensor, data conversion handle, data analysis, and calibration software. The special sensor I-scan 4000 was used for tibiofemoral joint. This sensor consists of two pieces of separate sensors, whose specifications are of 33 mm ∗ 28 mm, 0.1 mm thick. The I-scan 5051 sensor was used for patellofemoral joint's contact measurement [24]. In the experiments, the articular capsule was opened, and then the sensing piece of I-scan sensor was, respectively, put into the joint gaps of tibiofemoral and patellofemoral joint (Figure 7).

The gravity loading of the femoral head was controlled and* measured* by the sensor on the mobile beam of tension/compression testing machine. The quadriceps force and connection test device was connected to the force sensor CFBLS-25 (25 Kg maximum load, 0.03% FS, Shanghai Yichuan Instrument Factory) and the amplifier VM641 (±0.1% accuracy, Guangzhou Huamao Sensor Co., Ltd.). The sensor, pulley, and screw were connected to form loading and measuring device, which was used to measure and control the tension of quadriceps. The output signal of the amplifier was collected by the DAQ Card (data acquisition card) (Yanhua PCI-1710L, Advantech Co., Ltd.). And the measured results were outputted by the commercial software Labview (Laboratory Virtual Instrumentation Engineering Workbench, United States National Instruments Company).

#### 2.2.2. Experimental Loading and Measurement

**Figure 8:**
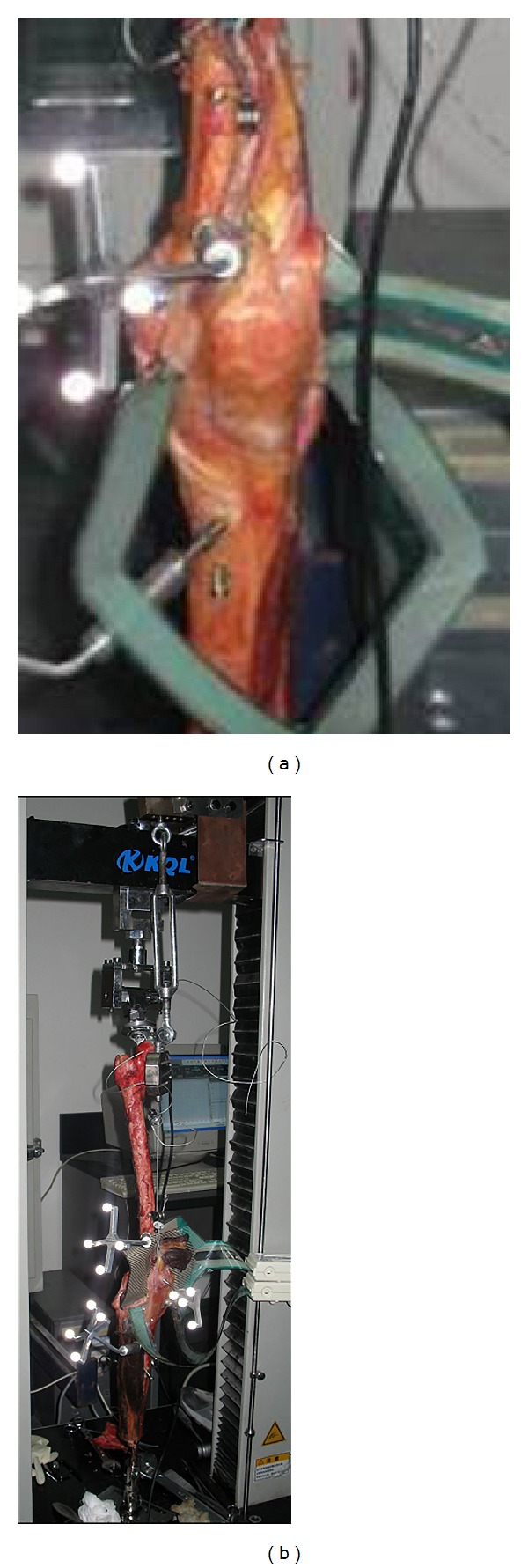
The device after installation.

The I-scan 4000 and the I-scan 5051 pressure test pieces were placed, respectively, in tibiofemoral and patellofemoral joint, and the articular capsule was sutured. The specimen was fixed into the experimental platform. The gravity loading on the femoral head was controlled and measured by sensor of tension/compression testing machine. The tension of quadriceps tendon was outputted by the DAQ Card and the commercial software Labview. Simultaneously, the contact pressure of tibiofemoral joint was recorded by the I-scan contact measurement system. For dynamic movement measurement, three reference frames consisting of 14 mm diameter markers were fixed in tibia, femur, and patella, respectively. The data of markers were captured and the relative motions of patella, tibia, and femur were measured by the Polaris optical tracking system (Figure 8).

## 3. Results

### 3.1. The FE Calculation and Experimental Results of Knee Joint

The FE model was developed into the software of ABAQUS-6.5.1 (HKS, Pawtucket, RI) to analyze the articular contact and the relative motion of the patellofemoral joint and tibiofemoral joint synchronously. By calculating the dynamic FEA of natural knee, the movement and joint contact stress of healthy knee could be obtained. The flexion motion and dynamic contact characteristics of knee joint were analyzed and verified by comparison with the data from cadaver in vitro experiment.

#### 3.1.1. The FE Results and Verification Analysis of Tibiofemoral Joint

**Figure 9:**
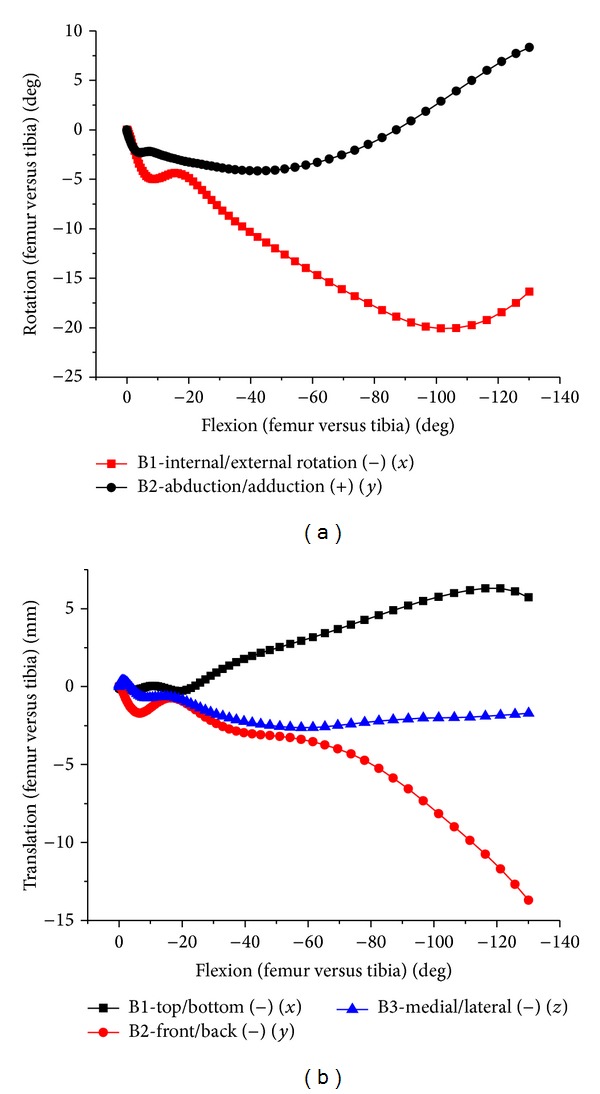
The relative movement of natural tibiofemoral joint. (a) Internal/external and abduction/adduction of tibiofemoral joint. (b) Translation of tibiofemoral joint.

**Figure 10:**
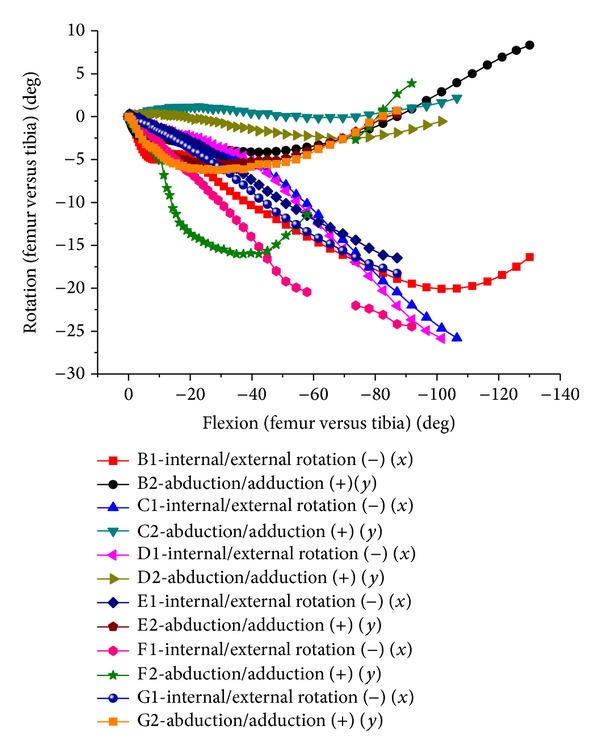
Internal/external and abduction/adduction of tibiofemoral joint relative movements in both test and FEA.

**Figure 11:**
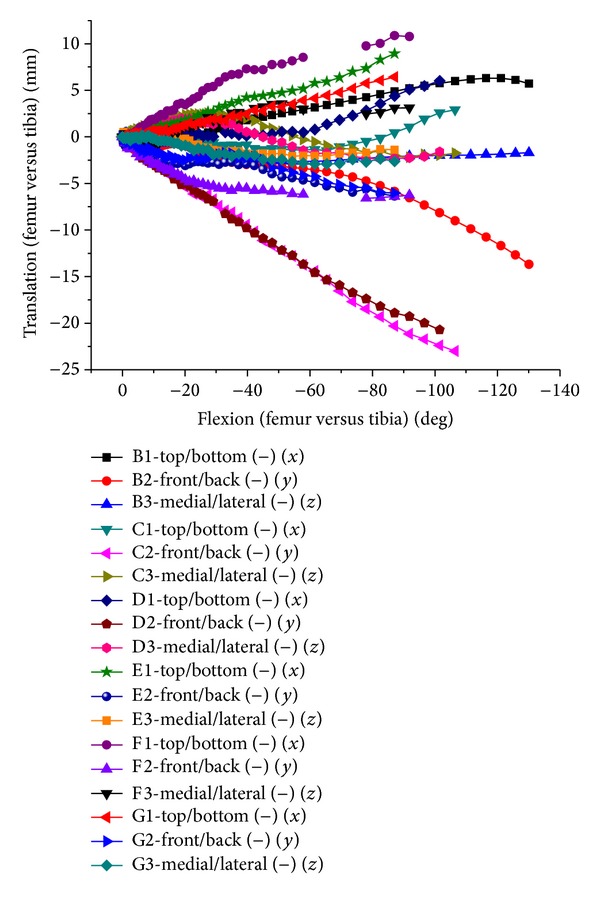
The relative translation of tibiofemoral joint in both test and FEA.

The tibial internal rotation increases as knee flexes, and femoral relative external rotation decreases after about 90° flexion, accompanying 9° adduction of femur (Figure 9). The femur backward translation increased along with knee flexing. In high flexion, femoral condyle lifted off tibial surface a few millimeters and contacted with posterior meniscus. Smaller medial-lateral translation happened in the entire flexion. The cadaver experiment and FE results were compared in contrast diagram (Figures 10 and 11).

During 0–90-degree knees flexion, femur external rotation was average of 20 degrees, and femur abduction turned to average of 2 degrees. From 90–120-degree flexion, the tibial internal rotation increased while femur external rotation decreased. Synchronously, the femoral adduction increased. There was unusual abduction for one of the specimens during earlier flexion stage. The femur translation trends of the experiment and FE results were basically consistent (Figure 11) in the direction of medial-lateral, up-down, and front-back, respectively. With flexion growth, femoral relative tibial translated upward, inward and backward, respectively. Two cases translated downward within 20–70-degree flexion. The backward translation was relatively small, which was approximately 7 mm at 90-degree flexion, being similar to simulation results. There was a large backward translation for one case, approximately 23 mm around 110-degree flexion. There was bigger difference for translation result, which may be caused by the error of selecting the osseous marker points and the individual difference.

**Figure 12:**
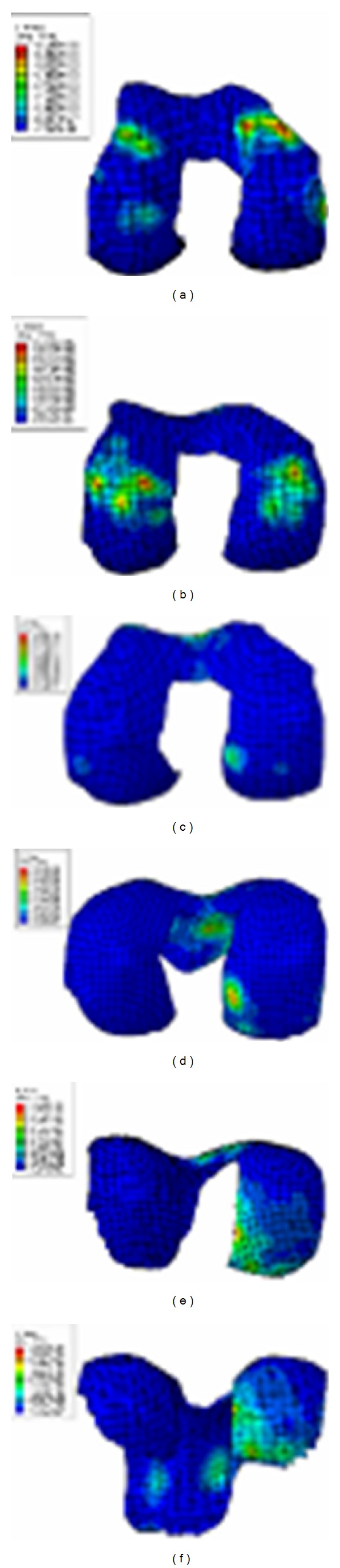
Contact stress of 0-30-60-90-130 degree flexion of tibiofemoral joint in FEA. (a) Stress distribution of 0° flexion. (b) Stress distribution of 30° flexion. (c) Stress distribution of 60° flexion. (d) Stress distribution of 90° flexion. (e) Stress distribution of 120° flexion. (f) Stress distribution of 130° flexion.

**Figure 13:**
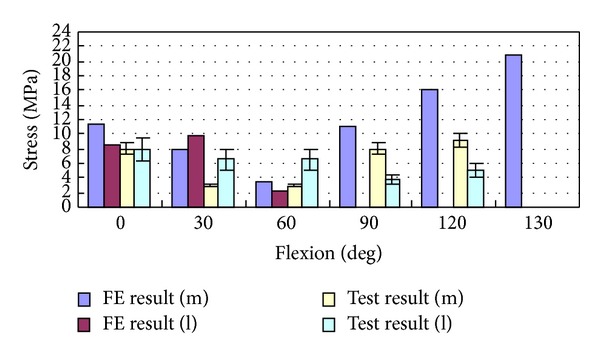
Comparison between experimentally measured stress applied to tibiofemoral joint and the FE results, where m refers to the medial femur and l refers to the lateral femur.

With knee flexion from 0 to 130 degrees, the average peak stress of tibiofemoral joint was 10 MPa at 0 degrees and 6 MPa at 30~90 degrees, and it increased to 21 MPa from 90 to 130 degrees. There were differences between the medial and the lateral tibiofemoral joint. From 0- to 60-degree flexion, medial and lateral contact stresses were close for FE results, but the lateral contact stress was higher than the medial contact stress for in vitro test. Starting from 90-degree flexion, larger contact stress was in the medial tibiofemoral joint for both of FE and in vitro test. Simultaneously, in the lateral tibiofemoral joint, there was little contact for FE results and about 4 MPa small contact stress for in vitro test. In 0–30-degree flexion, the contact of tibiofemoral joint mainly occurred in the front of tibia, and contact area was relatively small. Within 30~60-degree flexion, contact was mainly in the central tibia; contact area is increasing and causing smaller contact stress. With flexion deepening, the backward translation of femur increased, contacting with the posterior meniscus, and lateral femoral condyle lift off tibial surface a few millimeters in higher flexion (Figures 12 and 13). Accordingly, the total contact area of tibiofemoral joint relatively decreased, causing larger contact stress in the medial tibiofemoral joint.

#### 3.1.2. The FE Results and Verification Analysis of Patellofemoral Joint

**Figure 14:**
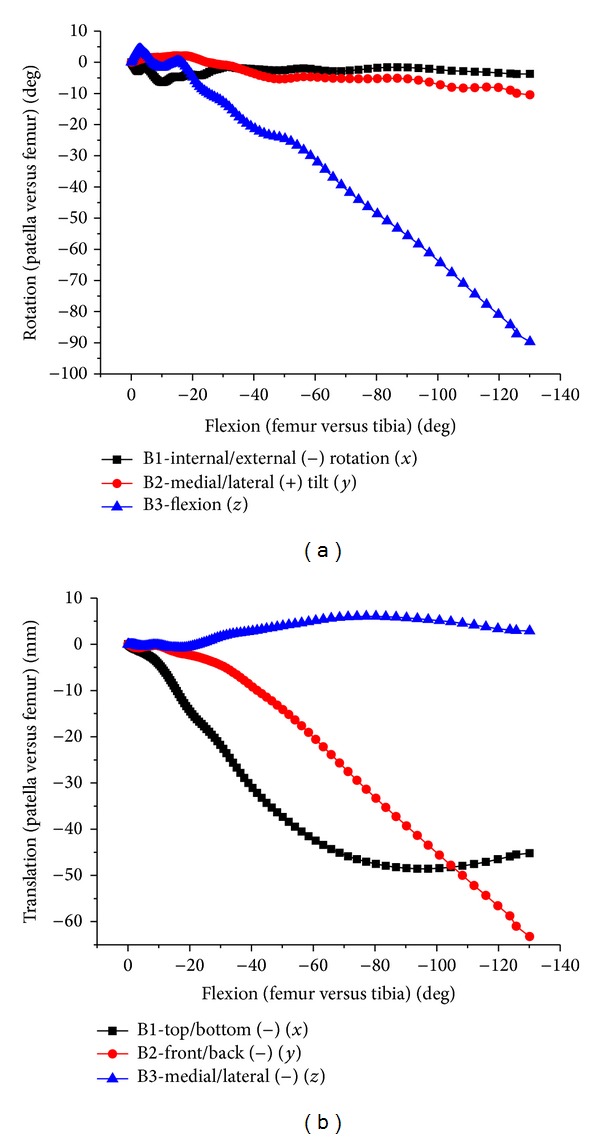
The relative movement of patellofemoral joint. (a) Internal/external, abduction/adduction, and flexion of patellofemoral joint. (b) Translation of patellofemoral joint.

With flexion of tibiofemoral joint, the flexion change of patellofemoral joint was basically linear (Figure 14). Accompanying patella's small angle external rotation and medial tilting relative to femur, there were small medial, backward, and upward translations. Patella was up to about 90 degrees flexion, external rotation of 3.7 degrees, medial tilting of 10 degrees, backward translation of 64 mm, the maximum downward translation of 49 mm at about 90 degrees knee flexion, and the maximum lateral translation of 6 mm at about 80 degrees knee flexion.

**Figure 15:**
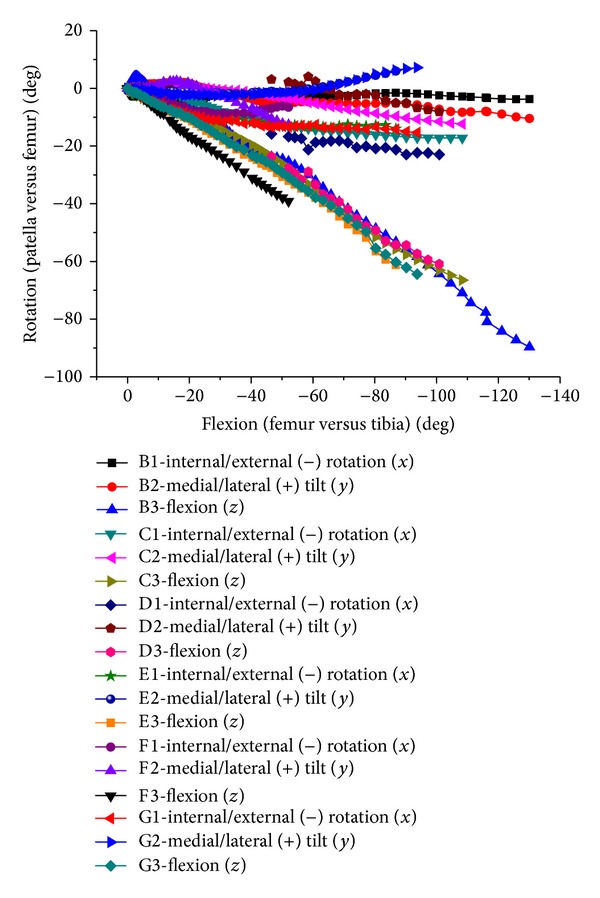
Internal/external, abduction/adduction, and flexion of patellofemoral joint relative movements in both test and FEA.

**Figure 16:**
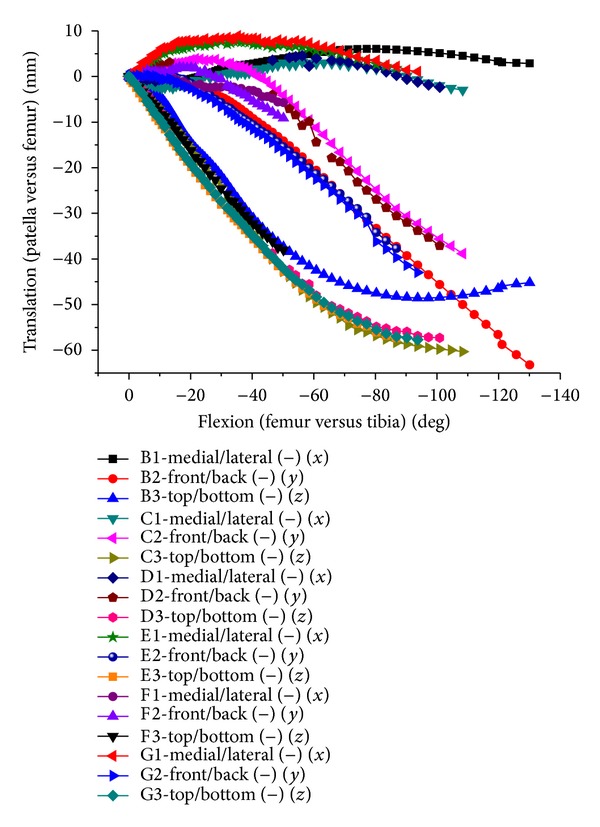
Translation of patellofemoral joint relative movement in both test and FEA.

The change tendency was basically consistent in simulation and test (Figures 15 and 16), being slightly different. The patella internal rotation (20 degrees maximum) was bigger than simulation results. There was little difference for patella relative medial tilting, excepting lateral tilting at 60 degrees flexion in one case. It was basically consistent in relative flexion of patellas, being linear with tibiofemoral flexion. The fluctuations of translation were larger than the rotation. The maximum medial translation in the experiment occurred within 30 degrees flexion earlier than the simulation of about 70 degrees flexion. However, the upward and backward translations were larger than the simulation. This difference may be caused by soft tissue relaxation in the cadaver experiment.

**Figure 17:**
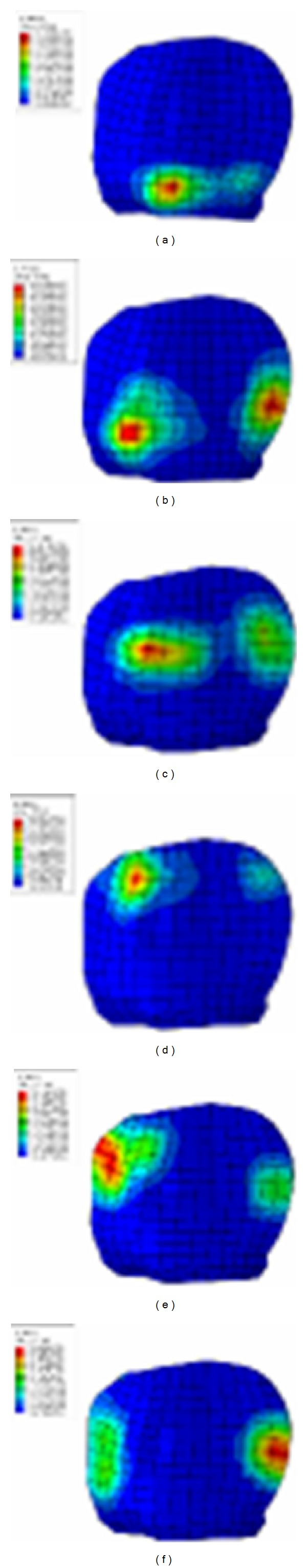
Contact stress of 0-30-60-90-130 degree flexion of tibiofemoral joint in FEA. (a) Stress distribution of 0° flexion. (b) Stress distribution of 30° flexion. (c) Stress distribution of 60° flexion. (d) Stress distribution of 90° flexion. (e) Stress distribution of 120° flexion. (f) Stress distribution of 130° flexion.

**Figure 18:**
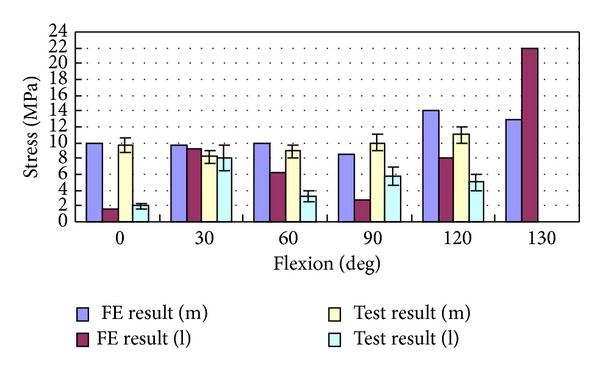
Comparison between experimentally measured stress applied to patella cartilage and the FE results, where m refers to the medial patella and l refers to the lateral patella.

With knee flexion, the contact place of patellofemoral joint gradually moved from the inferior patella to the superior patella and then turned slightly downward after 120 degrees. Within 30 degrees of flexion, the contact places of patellofemoral joint were unsteady. Within 30–90 degrees, the contact places of patellofemoral joint gradually drifted to the medial and lateral patella; the contact places were mainly on the medial patella ridge. Starting from 90-degree flexion, contact position was obviously distributed in medial and lateral margin patella. From 0- to 90-degree flexion, the contact stress was of about 9 MPa for both the simulation and the test results. The medial contact stress was significantly greater than the lateral one except within 30-degree flexion. The contact stress increased after 90°, reaching 22 MPa at 130-degree flexion. The margin patella contacted with femoral epicondylus in higher flexion and decreased contact area causing higher contact stress (Figures 17 and 18).

## 4. Discussion

### 4.1. The Experimental System Analysis

To fully understand the function of knee joint, not only the synchronal measurement of both tibiofemoral and patellofemoral articulations but also the synchronal measurement of both kinematics and contact can be implemented in this experiment system and simulation. Because of the difficulty of accurately measuring internal joint contact stress in rational functional conditions, there was seldom contact research about the tibiofemoral joint. Hsu et al. (1997) [25] used unidirectional sensor embedded in patellar prosthesis to measure patellofemoral joint contact force. And it was limited that the force and movement were measured separately. Powers et al. (1998) [26] used electromagnetic tracking equipment to measure the patella movement and used pressure sensitive film to measure patellofemoral joint contact pressure and area. However, tibiofemoral joint was not measured. Halloran et al. (2010) [9] developed a whole joint model, and this included fixture components only. Prior work with cadaveric specimens included displacement control of TF kinematics and only PF soft tissue representations [27].

The in vitro experiment and simulation results were compared in this study, and there were differences between them. The accuracy of test was inevitably influenced by some of the characteristics of experiment system. Although the thickness of sensing piece is only 0.1 mm, the joint surface shape was slightly changed, and the results accuracy of contact area and contact pressure peak in joint was affected. The difference of reference point selection caused the difference of coordinate system, which led to measurement error of movement. Due to the limitation of measurement mechanism itself, it is difficult to perfectly simulate the human knee joint squat movement. In vitro test research showed that quadriceps force size and loading direction influence tibiofemoral joint rotation, especially within 0~90 degrees flexion; the force and angle (Q angle) reduction of quadriceps caused tibial internal rotation reduction [28, 29]. Therefore, the difference between simulation and living quadriceps activities may be one of the reasons of the tibial rotation difference between in vitro and living tests. The signal interference of sensor, measurement errors of force transducer, contact stress sensor, reference frame, and calibration error can also cause measurement error. Because of losing activity, the muscles and joints of specimens will be stiff, and decreased joint fluid lubrication in articular capsule increased joint contact friction. And the mechanical property of bone and soft tissue changed accompanied with relaxing and drying of specimen organization itself. These conditions may all affect the experimental results. So there are differences between the simulation and the results of the experiment, as well as between the specimen and living body. In addition, individual differences can also cause difference. Therefore, the experimental method also needed to be continuously developed to improve the biomechanical research of knee.

### 4.2. Natural Human Knee Flexion Biomechanical Analysis

Along with the knee flexion, natural knee joint femur rolled backward and tibia internally rotated. The results of movement of femur relative tibia were basically consistent with Iwaki (2000) and Johal et al. (2005) [30, 31]. Within 0- to 30-degree flexion, tibial internal rotation increased (3.3–12.8 degrees). Within 30~90-degree flexion, natural knee tibial rotation was bigger (10.6–17.5), maintaining internal rotation, which is approximate to previous results of passive flexion with no loading (25-degree internal rotation at 100-degree passive flexion) [32]. And it was different from Hirokawa et al. (1992) [29] (1-degree external rotation at 120-degree flexion) and Van Kampen and Husikes (1990) [33] (internal rotation began until 120-degree flexion for three of the four specimens, in which fixed femoral test equipment was used). If rotating force was applied on tibia, especially for the unloading knee, femur external rotation at 90 degree is reversible. Therefore, if the tibia flexes 90 degrees at external rotation phase, it is possible to stretch to 20-degree flexion with no lateral condyle backward-forward translation. So, to great extent, the tibia rotation belongs to tibial rotation belong to “combining” not inevitable within 20~90 degrees flexion. However, the 20-degree flexed position was the critical point of tibia in internal rotation. Therefore, tibia internal rotation was inevitable within 0~20 degrees flexion, rotating around the long axis of knee.

The results of knee adduction-abduction about living body were little reported recently. The research in this paper showed that the femoral external rotation was along with the adduction. The reason is that the medial tibia platform is more concave than the lateral one and the projection on coronal plane of medial condyle is lower than that of the lateral condyle [34]. No eversion in knee flexion due to the consistent curvature radius of the medial and lateral femoral posterior condyle, no distal outstanding of medial condyle. The adduction in higher flexion was caused and corresponded with the internal rotation of tibia relative to femur. The research results of Hsieh et al. (1998) [35] (0-degree adduction at 90-degree flexion) and Kurosawa et al. (1985) [36] (2.2-degree adduction at 120-degree flexion) were within the scope of this study.

Within hol-extension to passive flexion (more than 120-degree flexion), there was 10 mm femoral condylar backward rolling. Femoral condyle scrolled up to meniscus posterior horn, almost dislocation to the tibia. This result is consistent with Nakagawa et al. (2000) [37] of no loading deep flexion Japan knee and Abdel-Rahman and Hefzy (1998) [38] ray research results. Wilson et al. (2000) [32] research results were of 24 ± 4 mm backward translation, 13 ± 4 mm distal translation, and 5 ± 3 mm medial translation after 100-degree flexion, which were approximate to this paper. Wretenberg et al. (2002) [39] analyzed 16 nonload right tibiofemoral joint contact at 0-degree, 30-degree, and 60-degree flexion by MRI and observed that the lateral displacement was bigger than the medial. The research of Johal et al. (2005) [31] showed that femoral condyle translated backward within 0~90-degree flexion, accompanied with slipping or rolling.

In this study, the distribution of medial and lateral contact stress was influenced by the joint position. The peak stress of tibiofemoral joint contact was of average 10 MPa at 0-degree flexion and 6 MPa within 30~90-degree flexion, and it grew from 90-degree flexion reaching 21 MPa at 130-degree flexion. In the flexion process, the medial and lateral tibiofemoral joint contact stresses were different. From 0- to 60-degree flexion, medial stress was approximate to the lateral stress for FEA and slightly larger contact stress at lateral joint for in vitro experiment. Within 0~30-degree flexion, contact position was mainly in anterior tibia, and contact area was relatively small. From 30- to 60-degree flexion, the contact mainly occurred in central tibia, increased contact area, and decreased contact stress. Along with deepening flexion, femur backward translation and liftoff increased, which decreased tibiofemoral articular contact area. After 90-degree flexion, larger contact stress occurred in medial joint for FEA and in vitro experiment. The 21 MPa maximum contact stress of simulation analysis results was closed to 25 MPa cartilage cracking limitation [40] (Torzilli et al., 1999), being consistent with Ashvin Thambyah et al. (2005) [5] which is of 14 MPa average stress peak (standard deviation 2.5 MPa) on walking phase. The loaded living research by Iwaki et al. (2000) and Hill et al. (2000) [30, 41] showed that large change occurred in high flexion for tibiofemoral joint, and too much stress (more than 25 MPa) caused cartilage injury, which may be the cause of the continued development of joint degeneration. Contact area reduction within high flexion was likely the reason of high stress in high flexion, and the cause of high stress was not only from high loading.

In lateral knee joint, considerable backward translation occurred, accompanied femur lifting off the lateral tibia and fall on the posterior horn of meniscus, the femur being subluxation. At the same time medial stress increased, which may be the reason of medial meniscus tear.

As for patellofemoral joint movement, Zavatsky et al. (2004) [42] gave the measurement result, and it is consistent with this research. Even though there are different reference points and reference frames, the calculation and measurement of patellofemoral relative rotation in this paper were within the standard deviation range being consistent with other research [35]. The patellofemoral joint translation motion results (backward, distal, medial, or lateral translation) also were approximate to other results [25, 33, 35]. At the same time, patellofemoral flexion was lagged behind the tibiofemoral flexion, which was consistent with the relative reports [33, 35, 43].

The contact point's upward movement of the healthy patellofemoral joint mainly occurred in early flexion, which was consistent with the previous in vitro [44, 45] and in vivo research [46]. The data of this study showed that, after 60-degree flexion, the contact area was relatively stable in the proximal portion of patella and symmetrically distributed in medial and lateral patellar surface, with no contact near the ridge of the patella. The research of Suzuki et al. (2012) [47] was approximate to this paper. And patellofemoral contact area increased with the knee flexion, of 70% average growth within 30 degrees flexion and of 34% average increase within 30~60 degrees flexion [48]. It was also found in this research that, with the deepening tibiofemoral joint flexion, the patellar tendon resulted in medial tilting so that the patella odd facet contact with the femur reduced the tension of the patellar tendon and quadriceps. At the same time, there was always a contact zone (concave area) at the upper patella and the contact occurred in the intercondylar notch along with the medial and lateral contact, a recess located in the upper patella exactly matching with the trochlear.

In higher flexion, the curvature radius of the femoral condyles was shorter and the lateral collateral ligament and anterior cruciate ligament were slack. Meanwhile, with tibiofemoral joint flexing, the tibiofemoral joint pressure increased, and the tension of the quadriceps tendon and the patellar tendon increased. The study showed that arthritis and cartilage damage was the result of the repeated or high contact stress [49], while the medial tibiofemoral was prone to arthritis, leading to an articulated knee. There was difference between Westerners and Asians, especially in the high flexion activities [50]. Arched knee is more likely to occur in Asians. It could not be ignored for the mechanical factors causing knee disease from the activities or ethnic differences between Asians and Westerners. Especially for Asians, the relationship between mechanics and movement in high flexion should be fully understood.
